# High PSQI score is associated with the development of dyskinesia in Parkinson’s disease

**DOI:** 10.1038/s41531-022-00391-y

**Published:** 2022-09-29

**Authors:** Xiaohui Tang, Jingyun Yang, Yining Zhu, Haiyan Gong, Hui Sun, Fan Chen, Qiang Guan, Lijia Yu, Weijia Wang, Zengping Zhang, Li Li, Guozhao Ma, Xijin Wang

**Affiliations:** 1 Department of Neurology, Shanghai Tongji Hospital, School of Medicine, Tongji University, Shanghai, China; 2 Department of Neurology,, Xinhua Hospital Affiliated to Shanghai Jiao Tong University School of Medicine, Shanghai, China; 3 Department of Neurology, Zhabei Central Hospital, Jing’an District, Shanghai, China; 4 Rush Alzheimer’s Disease Center, Rush University Medical Center, Chicago, IL USA; 5 Department of Neurological Sciences, Rush University Medical Center, Chicago, IL USA; 6 School of Mathematical Sciences, Fudan University, Yangpu District, Shanghai, China; 7 Department of Neurology, Suzhou BenQ Medical Center, The Affiliated BenQ Hospital of Nanjing Medical University, Suzhou, Jiangsu Province China; 8 Department of Neurology, Shanghai East Hospital, School of Medicine, Tongji University, Shanghai, China; 9 Department of Neurology, Shandong Provincial Hospital affiliated to Shandong First Medical University, Jinan, Shandong Province China

**Keywords:** Parkinson's disease, Risk factors

## Abstract

Dyskinesia is one of the most disabling motor complications in Parkinson’s Disease (PD). Sleep is crucial to keep neural circuit homeostasis, and PD patients often suffer from sleep disturbance. However, few prospective studies have been conducted to investigate the association of sleep quality with dyskinesia in PD. The objective of the current study is to investigate the association between sleep quality and dyskinesia and build a prediction model for dyskinesia in PD. We prospectively followed a group of PD patients without dyskinesia at baseline for a maximum of 36 months. Univariable and multivariable Cox regression with stepwise variable selection was used to investigate risk factors for dyskinesia. The performance of the model was assessed by the time-dependent area under the receiver-operating characteristic curve (AUC). At the end of follow-up, 32.8% of patients developed dyskinesia. Patients with bad sleep quality had a significantly higher proportion of dyskinesia compared with those with good sleep quality (48.1% vs. 20.6%, *p* = 0.023). Multivariable Cox regression selected duration of PD, sleep quality, cognition, mood, and levodopa dose. Notably, high Pittsburgh sleep quality index (PSQI) score was independently associated with an increased risk of dyskinesia (HR = 2.96, 95% CI 1.05–8.35, *p* = 0.041). The model achieved a good discriminative ability, with the highest AUC being 0.83 at 35 months. Our results indicated that high PSQI score may increase the risk of developing dyskinesia in PD, implying that therapeutic intervention targeting improving sleep quality may be a promising approach to prevent or delay the development of dyskinesia in PD.

## Introduction

Parkinson’s disease (PD) is the second common aging-related central neurodegenerative disease with different types of motor and non-motor symptoms. Levodopa, which can improve patients’ clinical symptoms including bradykinesia, rigidity, and akinesia, is considered to be the gold standard of PD treatment^1^. However, as the disease progresses, a majority of patients suffer from motor complications, including dyskinesia^2^. In 2012, Manson et al.^3^ reviewed studies of the incidence of dyskinesia in PD patients. They concluded that 40–50% of patients developed dyskinesia within 5 years since initiation of antiparkinsonian treatment and the percentage increased to 50–75% after 10 years. As one of the most disabling motor complications in PD patients, dyskinesia imposes a huge burden on the patients, their family, and society^4^.

Dyskinesia can have a variety of clinical forms including dystonic and choreic movements. It appears initially on the more affected body side at different points of a levodopa drug cycle^5^. The clinical manifestations of dyskinesia can be divided into three main patterns, including peak-dose dyskinesia, “off” dyskinesia, and biphasic dyskinesia^6^, with peak-dose dyskinesia being the most common in PD. Dyskinesia is believed to be associated with dysfunction of neural circuit^2,5,7^. Good sleep quality is crucial to maintain neural circuit homeostasis^8,9^. Many studies^10^ showed that PD patients usually suffered from sleep disturbance, such as long periods spent in bed while not asleep and unable to remain asleep and excessive daytime sleepiness. In addition, there is a growing body of evidence supporting that sleep disturbance may precede the development of neurodegenerative diseases, including PD^11–13^. A recent study found that an animal model of PD dyskinesia experienced a change in cortical slow-wave activity, which is of great importance to homeostasis during non–rapid eye movement sleep^14^. An in vivo study also showed that sleep deprivation aggravated dyskinesia and led to an earlier onset of dyskinesia^14^. However, few prospective studies have been conducted to explore the relationship between sleep quality and dyskinesia in PD patients.

Here, we prospectively followed a group of PD patients without dyskinesia at baseline for a maximum of 36 months to investigate the association between sleep quality and dyskinesia and build a prediction model for the development of dyskinesia.

## Results

### Patients’ clinical characteristics at the baseline

A total of 61 participants were included in this study. Characteristics of the study participants are presented in Table 1. The mean baseline age of the study cohort was 66.6 ± 6.1 years and the mean age of onset of PD was 61.6 ± 7.0 years. There were 41 (67.2%) men. The median duration of PD was 4.5 years (interquartile range [IQR] 2.9–7.8 years). The median MDS-UPDRS III was 22 (IQR 16.5–30.0). There were 27 (44.3%) patients who had motor fluctuation and 19 (31.1%) patients who had FOG. The median LEDD was 375.0 (IQR 275.0–611.9) mg and the median levodopa equivalent daily dose (LEDD) per kilogram body weight (LEDD/Weight) was 9.6 (IQR 6.6–12.9) mg/kg. The median Pittsburgh sleep quality index (PSQI) was 5.0 (IQR 3.0–9.5).

**Table 1:** Patients’ clinical characteristics at the baseline.

	Study participants (*N* = 61)
Age, y	66.6 ± 6.1
Male, No. (%)	41 (67.2%)
Age of onset of PD, y	61.6 ± 7.0
Education years, y	11.0 (8.0-14.0)
Duration of PD, y	4.5 (2.9–7.8)
H-Y	2.5 (1.8–3.0)
MDS-UPDRS III	22.0 (16.5–30.0)
NMSS score	234.0 (97.5–574.0)
HAMA score	6.0 (4.0–10.0)
HAMD score	7.0 (4.0–13.0)
MMSE score	29.0 (27.0–30.0)
MOCA score	27.0 (23.5–28.0)
Motor fluctuation No. (%)	27 (44.3%)
FOG, No. (%)	19 (31.1%)
LEDD, mg	375.0 (275.0–611.9)
LEDD/Weight, mg/kg	9.6 (6.6–12.9)
PSQI total	5.0 (3.0–9.5)
PSQI ≥ 6 No. (%)	27 (44.3%)
PDSS total	126.0 (114.5–134.0)
ESS total	6.0 (4.0–10.0)
RBD, No. (%)	30 (49.2%)

Results are expressed as mean ± standard deviation (normally distributed data) or median (interquartile range) (nonnormally distributed data).

*MDS-UPDRS III* part III of Movement Disorder Society Unified Parkinson’s Disease Rating Scale, *NMSS* Non-Motor Symptom Scale, *HAMA* Hamilton Anxiety Rating Scale, *HAMD* Hamilton Depression Rating Scale, *MMSE* Mini-Mental State Examination, *MOCA* Montreal Cognitive Assessment, FOG Freezing of Gait, *LEDD* levodopa equivalent daily dose, *PSQI* Pittsburgh Sleep Quality Index, *PDSS* Parkinson’s Disease Sleep Scale, *ESS* Epworth Sleepiness Scale, *RBD* Rapid Eye Movement Sleep Behavior Disorder Questionnaire.

### Incidence of dyskinesia during follow-up

**Fig. 1:**
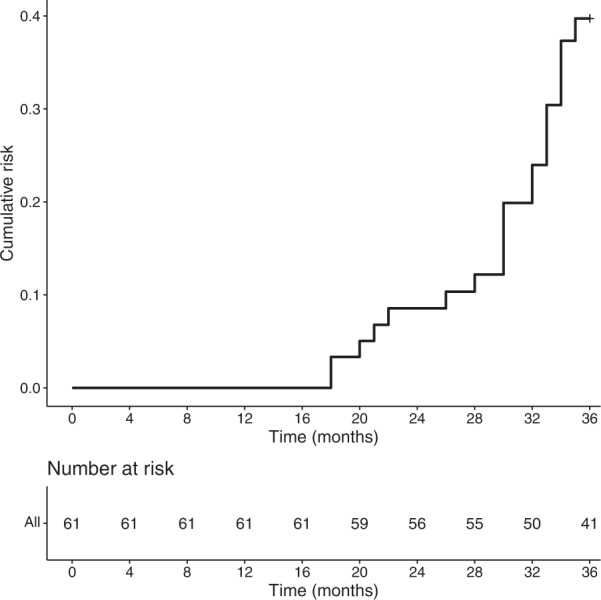
Kaplan–Meier estimates showing cumulative risk of dyskinesia in PD patients.

**Fig. 2:**
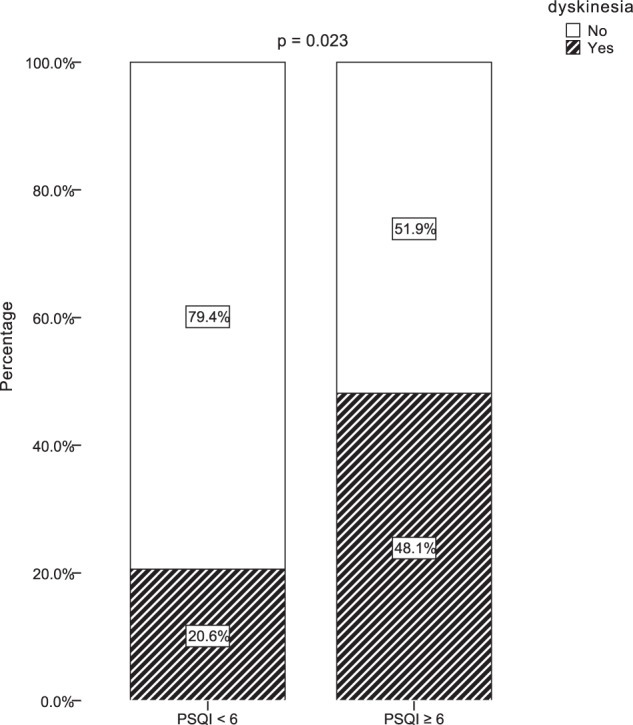
Incidence of dyskinesia at the end of follow-up. PSQI, Pittsburgh Sleep Quality Index.

During a median follow-up of 3.0 years (mean 2.8 years), 20 (32.8%) patients developed dyskinesia. The cumulative incidence of dyskinesia was 8.2%, 11.5%, 21.3%, and 32.8% at the 24-, 28-, 32-, and 35-month follow-up, respectively (Fig. 1). At the end of follow-up, patients with bad sleep quality had a significantly higher proportion of dyskinesia compared with those with good sleep quality (48.1% vs. 20.6%, *p* = 0.023) (Fig. 2).

### Risk factors of dyskinesia

**Fig. 3:**
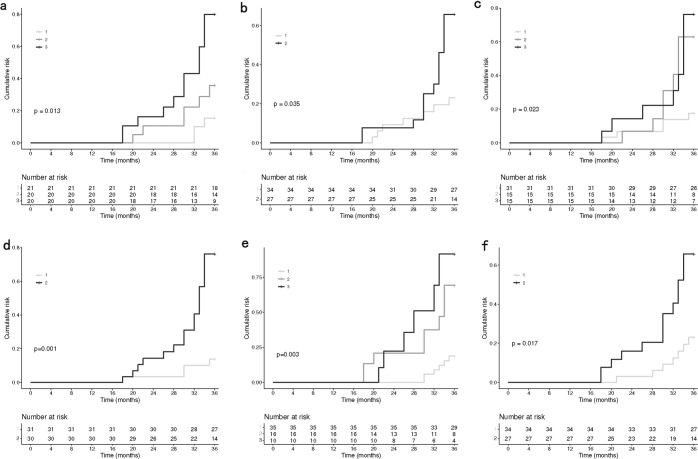
Kaplan–Meier estimates showing the effects on dyskinesia of some clinical characteristics. **a** NMSS (“1” represents the first tertile, “2” represents the second tertile, and “3” represents the third tertile), **b** HAMA (“1” represents <7, and “2” represents ≥7), **c** LEDD (“1” represents ≤400 mg, “2” represents 400~600 mg, and “3” represents >600 mg), **d** LEDD/Weight (“1” represents ≤median, and “2” represents >median), **e** duration of PD (“1” represents ≤5 y, “2” represents 5~10 y, and “3” represents > 10 y), **f** PSQI (“1” represents <6, and “2” represents ≥6). NMSS Non-Motor Symptom Scale, HAMA Hamilton Anxiety Rating Scale, LEDD Levodopa equivalent daily dose, PSQI Pittsburgh Sleep Quality Index.

Kaplan–Meier curves comparing the cumulative incidence of dyskinesia with respect to NMSS, HAMA, LEDD, LEDD/weight, duration of PD, and PSQI were shown in Fig. 3a–f. Kaplan–Meier curves comparing the cumulative incidence of dyskinesia with respect to MOCA, H-Y, and PDSS were shown in Supplementary Fig. 1a–c. We found that patients who had bad sleep quality had a significantly higher risk to develop dyskinesia than those who had good sleep quality (*p* = 0.017, Fig. 3f).

In addition, Cox proportional hazards analysis was conducted to examine risk factors for dyskinesia in PD (Table 2). In the univariable Cox analysis, long duration of PD was associated with a greater risk of developing dyskinesia (Hazard ratio; HR = 2.32, 95% confidence interval (CI) 1.38–3.92, *p* = 0.002). In addition, high LEDD (HR = 1.92, 95% CI 1.15–3.20, *p* = 0.012) and high LEDD/Weight (HR = 5.35, 95% CI 1.78–16.10, *p* = 0.003) also showed significant association with greater risk of dyskinesia. Furthermore, multiple non-motor clinical characteristics were associated with a greater risk of dyskinesia, including high NMSS score (HR = 2.29, 95% CI 1.27–4.16, *p* = 0.006), high HAMA score (HR = 2.64, 95% CI 1.05–6.62, *p* = 0.039) and high PSQI score (HR = 2.93, 95% CI 1.17–7.36, *p* = 0.022).

**Table 2:** Cox regression analyses of dyskinesia (*N* = 61).

Variables	Univariable analysis			Multivariable analysis		
	*β*	HR (95% CI)	*p*	*β*	HR (95% CI)	*p*
Age	−0.125	0.88 (0.36–2.17)	0.785	NA	NA	NA
Male	−0.436	0.65 (0.24–1.78)	0.398	NA	NA	NA
Duration of PD	0.843	2.32 (1.38–3.92)	**0.002**	0.736	2.09 (1.05–4.17)	**0.037**
Motor fluctuation	0.746	2.11 (0.86–5.16)	0.103	NA	NA	NA
FOG	0.707	2.03 (0.84–4.90)	0.125	NA	NA	NA
RBD	0.469	1.60 (0.65–3.91)	0.305	NA	NA	NA
H-Y	0.839	2.31 (0.95–5.67)	0.067	NA	NA	NA
MDS-UPDRS III	0.050	1.68 (0.70–4.06)	0.248	NA	NA	NA
NMSS	0.830	2.29 (1.27–4.16)	**0.006**	NA	NA	NA
HAMA	0.970	2.64 (1.05–6.62)	**0.039**	NA	NA	NA
HAMD	0.274	1.32 (0.53–3.30)	0.559	−0.978	0.38 (0.12–1.17)	0.090
MMSE	0.571	1.77 (0.52–6.05)	0.363	NA	NA	NA
MOCA	0.764	2.15 (0.89–5.19)	0.090	1.486	4.42 (1.40–14.00)	**0.011**
LEDD	0.654	1.92 (1.15–3.20)	**0.012**	NA	NA	NA
LEDD/Weight	1.680	5.35 (1.78–16.10)	**0.003**	1.120	3.06 (0.93–10.10)	0.064
PSQI	1.080	2.93 (1.17–7.36)	**0.022**	1.085	2.96 (1.05–8.35)	**0.041**
PDSS	1.080	2.94 (0.86–10.10)	0.087	NA	NA	NA
ESS	0.226	1.25 (0.50–3.14)	0.630	NA	NA	NA

*FOG* Freezing of Gait, *RBD* Rapid Eye Movement Sleep Behavior Disorder Questionnaire, *MDS-UPDRS III* part III of Movement Disorder Society Unified Parkinson’s Disease Rating Scale, *NMSS* Non-Motor Symptom Scale, *HAMA* Hamilton Anxiety Rating Scale, *HAMD* Hamilton Depression Rating Scale, *MMSE* Mini-Mental State Examination, *MOCA* Montreal Cognitive Assessment, *LEDD* levodopa equivalent daily dose, *PSQI* Pittsburgh Sleep Quality Index, *PDSS* Parkinson’s Disease Sleep Scale, *ESS* Epworth Sleepiness Scale, *HR* Hazard Rati, *CI* Confidence Interval, *NA* no applicable.

Bold fonts indicate statistical significance at *p* < 0.05.

**Fig. 4:**
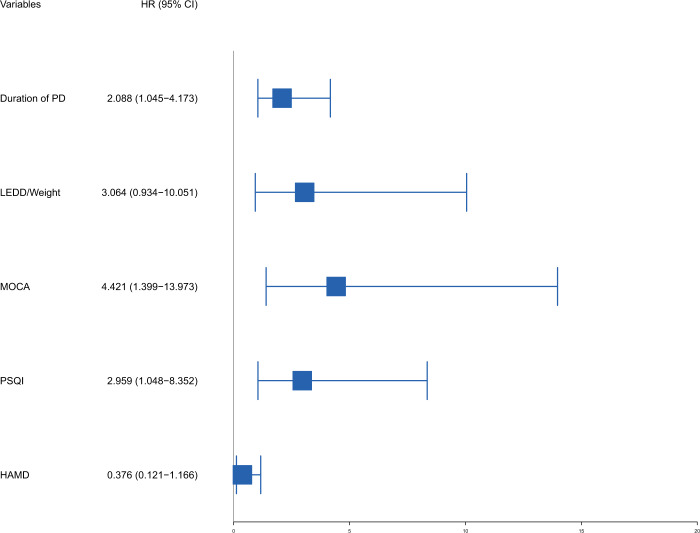
Forest plot of multivariable Cox regression analysis. LEDD Levodopa equivalent daily dose, MOCA Montreal Cognitive Assessment, PSQI Pittsburgh Sleep Quality Index, HAMD Hamilton Depression Rating Scale.

**Fig. 5:**
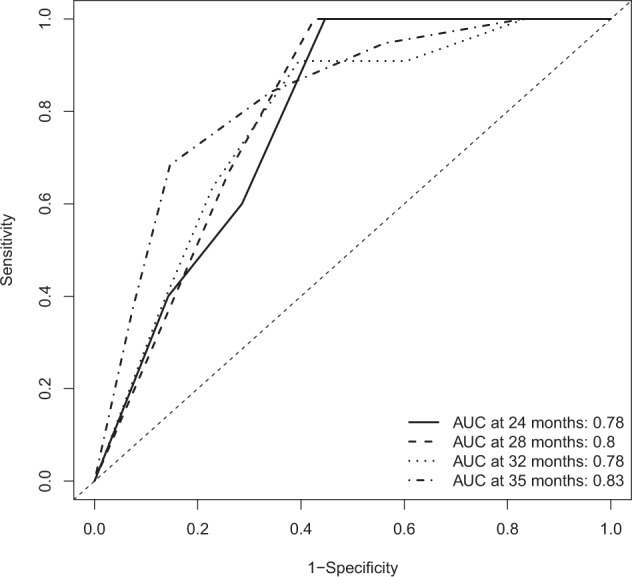
Time-dependent receiver-operating curve for prediction of dyskinesia in patients with Parkinson’s disease. AUC Area under curve.

The results of the multivariable Cox regression analysis were shown in Table 2. Forest plot of the multivariable Cox regression analysis was displayed in Fig. 4. We found that high PSQI was independently associated with the risk of dyskinesia (HR = 2.96, 95% CI 1.05–8.35, *p* = 0.041). In addition, long duration of PD (HR = 2.09, 95% CI 1.05–4.17, *p* = 0.037), and low MOCA score (HR = 4.12, 95% CI 1.40–14.00, *p* = 0.011) also showed significant association with dyskinesia. However, the association of high LEDD/Weight (HR = 3.06, 95% CI 0.93–10.10, *p* = 0.064) and high HAMD score (HR = 0.38, 95% CI 0.12–1.17, *p* = 0.090) with dyskinesia were of borderline significance. To evaluate the performance of the Cox model, we generated time-dependent ROC curves (Fig. 5). The area under receiver-operating characteristic curve (AUC) was 0.78, 0.80, and 0.78 at 24 months, 28 months, and 32 months since baseline, respectively, with the highest AUC being 0.83, which was reached at 35 months since baseline.

## Discussion

In this study, we prospectively followed a group of patients without dyskinesia at baseline for a maximum of 36 months. We found that a high PSQI score was independently associated with the development of dyskinesia and the predictive model achieved a good discriminative ability. These results highlight the important effect of sleep quality on the risk of dyskinesia and imply that therapeutic intervention targeting improving sleep quality may be a promising approach to prevent or delay the development of dyskinesia in PD.

Disrupted slow-wave sleep could accelerate the progression of PD^15,16^. Prior research indicated that sleep disturbance might precede motor symptoms in PD^13,17^. It is widely believed that slow homeostatic adjustment of intrinsic excitability occurring during sleep is fundamental for the stabilization of the network by gradual modification of plasticity thresholds^14,18^. Galati et al.^14^ conducted an assessment on synaptic downscaling across sleep episodes in a parkinsonian rat model showing dyskinetic movements similar to levodopa-induced dyskinesia. They observed a synaptic homeostasis impairment during sleep in rats showing dyskinesia^14^. Moreover, sleep deprivation led to anticipation and aggravation of dyskinesia in rats, supporting an association between sleep disturbance and the development of dyskinesia^14^. In recent years, a bulk of evidence has shown that dyskinesia is associated with neurodegeneration in cortical and subcortical areas, including the prefrontal cortex, primary motor cortex, striatum, subthalamic nucleus, and cerebellum^4^. Cortical activity was observed to be dominated by spindles and slow waves during sleep^19–21^. Sleep could enable a downward firing rate to maintain homeostasis and overall sleep slow-wave activity potentiates the majority of cortical synaptic plasticity^22,23^, implying that sleep disturbance could disrupt synaptic plasticity or synaptic homeostasis^24^. As a result, bad sleep quality may increase the risk of developing dyskinesia in PD by interrupting neural circuit homeostasis^25^. These findings, together with ours, provide important clues for further investigation of potential treatments of dyskinesia.

Previous research reported that disease duration and dose of levodopa were important etiopathogenic factors for LID^25^. In this study, the duration of PD was confirmed as a predictor for dyskinesia, while the association of LEDD/Weight with dyskinesia was of borderline significance in the multivariable Cox regression analysis, probably due to limited statistical power resulting from the limited sample size of our study. Cumulative LEDD^26^ was found to be a risk factor for LID, indicating that levodopa use, especially in a day-night pulsatile manner, could be a cause of subsequently aggravated LID^22^. Nevertheless, levodopa is necessary but not enough to generate LID. Dyskinesia is caused by pre-synaptic nigro-striatal degeneration and deteriorates along with disease duration. It is also associated with a relatively spared post-synaptic nigro-striatal system^27^, as supported by a prior study^28^ showing that dyskinesia development was a function of disease duration. Moreover, previous studies supported that long-term levodopa treatment for PD resulted in dyskinesia, suggesting a sleep-dependent depotentiation impairment in the dorsal striatum^29^. Future therapeutic strategies for PD may benefit from targeting sleep-dependent depotentiation impairment. Interestingly, a case-control polysomnographic study showed that reduced total sleep time was associated with increased LEDD and levodopa use could be a cause of reduced total sleep time^22^. These results highlight the importance of further investigate the relationship between sleep disturbance and dyskinesia.

In our study, we found that cognitive impairment as assessed by the global MOCA score was also associated with dyskinesia. Indeed, the reduction of the connectivity between the left inferior frontal cortex and the right motor cortex has been reported in dyskinesia patients^30^. It was speculated that decreased inhibitory action of the prefrontal cortex could produce disinhibition in both motor and cognitive control loops in PD^7^. Further studies with functional magnetic resonance and scales of different part of cognition in larger samples are needed to explore the association between cognitive impairment and dyskinesia.

To assess the relationship between change of PSQI and change of other clinical characteristics, we also calculated their annual change and the corresponding correlation (Table 3). The annual change of PSQI was ~0 (IQR ~0–0.343). The annual change of PSQI was associated with worsened non-motor symptoms, greater anxiety, worsened cognition, and increased levodopa dose, suggesting that patients experienced more impaired mood disturbance and cognitive dysfunction as sleep quality deteriorated. These findings are consistent with previous studies which observed a strong association between sleep disturbance and subsequent progression of PD^31,32^ and rapid motor progression in patients with rapid eye movement sleep behavior disorder (RBD). The exact pathophysiology of the association of sleep disturbance with dyskinesia needs to be explored in future studies.

**Table 3:** Correlation between the annual change of PSQI and annual changes of other clinical parameters.

Annual change of clinical parameters	Annual change of PSQI	
	*r*	*p*
Duration of PD	−0.101	0.422
H-Y	0.162	0.212
MDS-UPDRS III	0.162	0.212
NMSS	0.665	**<0.001**
HAMA	0.528	**<0.001**
HAMD	0.249	0.053
MMSE	0.110	0.399
MOCA	0.280	**0.029**
LEDD, mg	0.399	**0.002**
LEDD/Weight, mg/kg	0.413	**<0.001**
PDSS	−0.660	**<0.001**
ESS	0.658	**<0.001**

*MDS-UPDRS III* part III of Movement Disorder Society Unified Parkinson’s Disease Rating Scale, *NMSS* Non-Motor Symptom Scale, *HAMA* Hamilton Anxiety Rating Scale, *HAMD* Hamilton Depression Rating Scale, *MMSE* Mini-Mental State Examination, *MOCA* Montreal Cognitive Assessment, *LEDD* levodopa equivalent daily dose, *PSQI* Pittsburgh Sleep Quality Index, *PDSS* Parkinson’s Disease Sleep Scale, *ESS* Epworth Sleepiness Scale.

Bold fonts indicate statistical significance at *p* < 0.05.

There are some limitations of this study. First, the assessments of dyskinesia and sleep were conducted with subjective scales, which might suffer from subjective assessment bias. However, these scales are convenient, reliable, and practicable for clinicians. In addition, to minimize biases, each patient was assessed by two raters in a randomized and assessor-masking way. Second, the sample size of our study is limited, preventing us to match important characteristics other than PQSI to estimate more precisely the effect of PSQI on the risk of dyskinesia. As a result, the findings of this study need to be validated in future studies of large sample sizes, especially studies that match important clinical characteristics that might affect the risk of dyskinesia. Despite these limitations, the present work was among the few prospective studies to examine the relationship between dyskinesia and sleep disturbance, with a relatively long follow-up.

In summary, the current study found that a high PSQI score was a risk factor for the development of dyskinesia in patients with PD, suggesting that therapeutic intervention targeting sleep quality may be a promising approach to prevent or delay the development of dyskinesia in PD. The findings of our study need to be validated and the pathophysiological mechanisms underlying the findings need to be explored. Indeed, we are planning a new cohort study with larger sample size and a new project utilizing animal models to validate our findings and explore the pathological mechanisms.

## Methods

### Participants

**Fig. 6:**
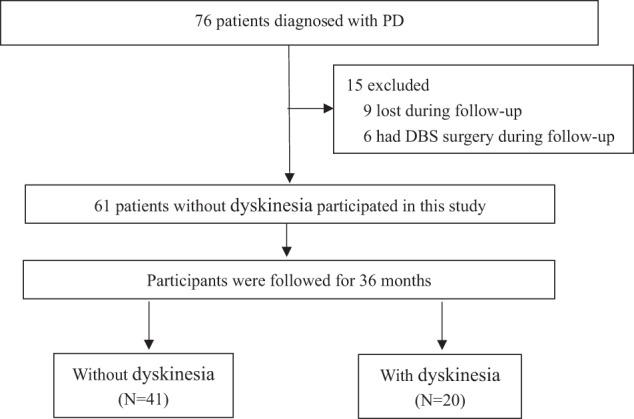
Flow diagram of the study. DBS deep brain stimulation, PD Parkinson’s disease.

A total of 76 patients were diagnosed with PD by experienced experts in the Department of Neurology, Shanghai Tongji Hospital, School of Medicine, Tongji University and Department of Neurology, Xinhua Hospital Affiliated to Shanghai Jiao Tong University School of Medicine based on the Unified Kingdom PD Society Brain Bank Clinical Diagnostic Criteria^33^. Exclusion criteria included: atypical and secondary parkinsonism, sleep apnea syndrome, severe mental diseases, never taken levodopa, a history of deep brain stimulation surgery, and unable to complete clinical evaluation due to cognitive impairment. Of the 76 patients, 15 patients were excluded and 61 patients who were without dyskinesia at the baseline were prospectively followed (Fig. 6). These subjects all received levodopa medications regularly for at least one month^22^. All patients were followed through outpatients visit in a blinded manner every three months until the onset of dyskinesia or the end of 36 months. This study was approved by the Ethics Committees of Shanghai Tongji Hospital, School of Medicine, Tongji University, and Xinhua Hospital Affiliated with Shanghai Jiao Tong University School of Medicine. Written informed consent was obtained from all patients.

### Assessments of demographic and clinical characteristics

Demographic and clinical characteristics include age, sex, age of PD onset, years of education, duration of PD, and weight. All patients’ motor symptoms were evaluated by the Movement Disorder Society–sponsored revision of the Unified Parkinson Disease Rating Scale III (MDS-UPDRS III) and H-Y stage. Levodopa dose was defined as the dose taken at the time of the first visit. Total LEDD was calculated based on the established algorithm^34^. Global non-motor symptoms were evaluated by the non-motor symptoms scale (NMSS). Cognition was evaluated by the Mini-Mental State Examination (MMSE) and the Montreal Cognitive Assessment (MOCA, score ≥26 was used as the reference in Cox regression analysis in this study). Mood complaints were investigated by the Hamilton Anxiety Rating Scale (HAMA) and the Hamilton Depression Rating Scale (HAMD). Patients’ quality of life was assessed by the Parkinson’s Disease Questionnaire-8 (PDQ-8). Freezing of gait (FOG) was evaluated by the New Freezing of Gait Questionnaire^35^. Annual change of clinical characteristics was calculated. For example, the annual change in HAMD was calculated as (follow-up HAMD—the baseline HAMD)/time of follow-up (year).

### Assessments of dyskinesia and sleep

Dyskinesia was assessed by two raters blinded to patients’ sleep based on the Movement Disorder Society-sponsored revision of the Unified Parkinson Disease Rating Scale IV^22,36^. If evidence for determining dyskinesia came from patients or their caregivers, they need to provide the raters with related videos. It is worth noting that the PD patients who developed dyskinesia during the follow-up were all peak-dose dyskinesia rather than biphasic dyskinesia or “off” dyskinesia. Four sleep scales were used to assess sleep functioning, including the PSQI^37^, the Epworth Sleepiness Scale (ESS)^38^, the Rapid Eye Movement Sleep Behavior Disorder Questionnaire—Hong Kong (RBDQ-HK)^39,40^, and the Parkinson’s Disease Sleep Scale (PDSS)^41^. PSQI, the most commonly used measurement tool for sleep quality in clinical application, was designed to assess general sleep quality over the past month in clinical populations^37^. It has 19 subitems categorized into seven components, and score was summed to obtain a global score. According to conventions in previous studies, patients were dichotomized as having bad (PSQI ≥ 6) and good (PSQI < 6) sleep quality^42–44^. Other continuous variables were converted to categorical ones when necessary, with cutoff being set based on previous conventions or the median value in the total sample (Fig. 3a–f and Supplementary Fig. 1a–c).

In our study, all patients were evaluated in the “on” state. All raters received homogeneity training at the start of the study to reduce inter-rater disagreement.

### Statistical analysis

Continuous data were presented as mean ± standard deviation or median (IQR) and compared by using independent *t* test or Mann–Whitney *U* test, as appropriate. Categorical variables were presented as numbers and percentages and compared by Pearson’s chi-square or Fisher’s exact test, as appropriate. Cumulative incidence of dyskinesia was displayed using Kaplan–Meier curves with log-rank test to compare the differences. Cox proportional hazards regression was applied to explore risk factors for developing dyskinesia. We conducted univariable Cox models for all baseline variables, followed by stepwise variable selection to select variables for the multivariable Cox regression, with the significance level for entry and stay being 0.1 (the default setting) to avoid rejection of potentially important variables due to uncontrolled confounders. HR with 95% CI was calculated. This was done using the *my.stepwise.coxph* in the *My.stepwise* R package. Multicollinearity of the selected variables was assessed using the variance inflation factor (VIF) (Supplementary Table 1). The assumption of proportional hazard was tested using scaled Shoenfeld residuals. The performance of the multivariable Cox model was assessed using a time-dependent area under the receiver-operating characteristic (ROC) curve (AUC).

A two-sided *p* value < 0.05 was considered to be statistically significant. Statistical analysis was performed using R version 4.2.0 (Foundation for Statistical Computing, Vienna, Austria).

### Reporting summary

Further information on research design is available in the Nature Research Reporting Summary linked to this article.

## Supplementary information

Supplementary Figure 1 and Table 1

## Data Availability

The data that support the findings of this study are available from the Department of Neurology, Shanghai Tongji Hospital, School of Medicine, Tongji University, via the corresponding authors.

## Code availability

R codes for data analysis are available upon reasonable request.
