# Supplementary material for: High PSQI score is associated with the development of dyskinesia in Parkinson’s disease
Source: NPJ Parkinsons Dis. 2022 Sep 29;8:124. doi: 10.1038/s41531-022-00391-y (PMC9522669; doi:10.1038/s41531-022-00391-y)
Supplement: Supplementary file 1 — Supplementary Figure 1 and Table 1 [file 41531_2022_391_MOESM1_ESM.pdf]

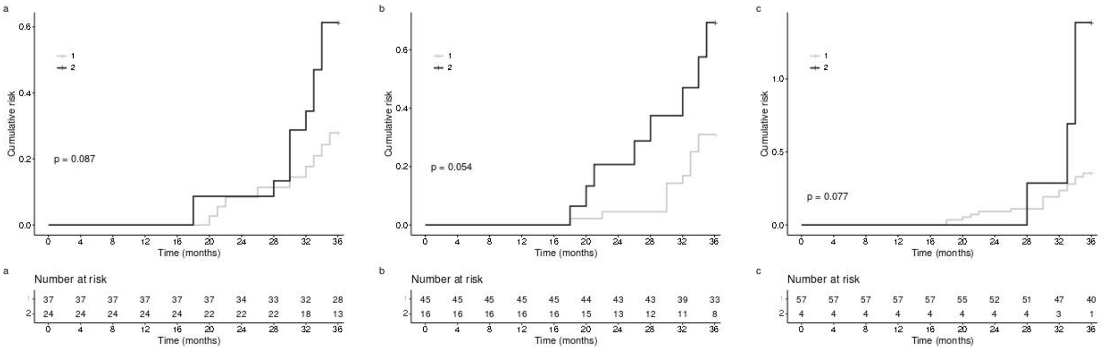

Supplementary Figure 1. Kaplan–Meier estimates showing the effects on dyskinesia of some clinical characteristics. (a) MOCA ("1" represents ≥ 26, and "2" represents < 26), (b) H-Y ("1" represents ≤ 2.5, and "2" represents > 2.5), (c) PDSS ("1" represents ≥ 90, and "2" represents < 90). MOCA, Montreal Cognitive Assessment; PDSS, Parkinson's Disease Sleep Scale.

Supplementary Table 1. Variance inflation factor of the selected variables in the multivariable Cox regression.

Variables	Duration of PD	LEDD/Weight	MOCA	PSQI	HAMD
Variance inflation factor	1.341	1.211	1.370	1.278	1.281

Abbreviations: *LEDD*, L-dopa equivalent daily dose; *MOCA*, Montreal Cognitive Assessment; *PSQI*, Pittsburgh Sleep Quality Index; *HAMD*, Hamilton Depression Rating Scale.
